# An oncogenic *CTNNB1* mutation is predictive of post-operative recurrence-free survival in an *EGFR*-mutant lung adenocarcinoma

**DOI:** 10.1371/journal.pone.0287256

**Published:** 2023-06-22

**Authors:** Yeseul Kim, Bokyung Ahn, Shinkyo Yoon, Goeun Lee, Deokhoon Kim, Sung-Min Chun, Hyeong-Ryul Kim, Se Jin Jang, Hee Sang Hwang

**Affiliations:** 1 Department of Pathology, Korea University College of Medicine, Korea University Anam Hospital, Seoul, Republic of Korea; 2 Department of Pathology, University of Ulsan College of Medicine, Asan Medical Center, Seoul, Republic of Korea; 3 Department of Oncology, University of Ulsan College of Medicine, Asan Medical Center, Seoul, Republic of Korea; 4 Department of Thoracic and Cardiovascular Surgery, University of Ulsan College of Medicine, Asan Medical Center, Seoul, Republic of Korea; Guangdong Medical University, CHINA

## Abstract

The Wnt/β-catenin pathway is known to be frequently dysregulated in various human malignancies. Alterations in the genes encoding the components of Wnt/β-catenin pathway have also been described in lung adenocarcinoma. Notably however, the clinical impacts of Wnt/β-catenin pathway alterations in lung adenocarcinoma have not been fully evaluated to date. We here investigated the prognostic implications of single gene variations in 174 cases of surgically resected lung adenocarcinoma tested using targeted next-generation sequencing. Screening of the prognostic impact of single gene alterations identified an association between *CTNNB1* mutation and poor recurrence-free survival in *EGFR*-mutant LUADs. Based on these results, the entire cohort was stratified into three groups in accordance with the mutational status of Wnt/β-catenin pathway genes (i.e. oncogenic *CTNNB1* mutation [*CTNNB1*-ONC], other Wnt/β-catenin pathway gene mutations [Wnt/β-catenin-OTHER], and wild type for Wnt/β-catenin pathway genes [Wnt/β-catenin-WT]). The clinicopathologic characteristics and survival outcomes of these groups were then compared. Oncogenic *CTNNB1* and other Wnt/β-catenin pathway gene mutations were identified in 10 (5.7%) and 14 cases (8.0%), respectively. The *CTNNB1*-ONC group cases displayed histopathologic features of conventional non-mucinous adenocarcinoma with no significant differences from those of the other groups. Using β-catenin immunohistochemistry, we found that the *CTNNB1*-ONC group displayed aberrant nuclear staining more frequently, but only in 60% of the samples. The LUADs harboring a *CTNNB1*-ONC exhibited significantly poorer RFS outcomes than the other groups, regardless of the β-catenin IHC status. This was a pronounced finding in the *EGFR*-mutant LUADs only in subgroup analysis, which was then confirmed by multivariate analysis. Nevertheless, no significant OS differences between these Wnt/β-catenin groups were evident. Hence, oncogenic *CTNNB1* mutations may be found in about 6% of lung adenocarcinomas and may predict post-operative recurrence in *EGFR*-mutant LUADs. Aberrant nuclear β-catenin staining on IHC appears to be insufficient as a surrogate marker of an oncogenic *CTNNB1* mutation.

## Introduction

Lung cancer is one of the leading causes of cancer-related deaths worldwide [1]. Lung adenocarcinoma (LUAD) is the most common histologic subtype, accounting for almost 70% of all lung cancers [2]. The identification of oncogenic mutations in LUADs has contributed to our understanding of the pathogenesis of lung cancer, helped to predict recurrence and prognosis, and contributed to the development of therapeutic target agents [3, 4]. Β-catenin, a protein encoded by the *CTNNB1* gene, is the main intracellular transducer of the canonical Wnt pathway that plays significant roles in tissue homeostasis and embryonic development [5]. Activation of the canonical Wnt pathway in tumor cells causes the nuclear accumulation of β-catenin, which regulates gene transcription and other cellular processes [5]. The nuclear translocalization of β-catenin accelerates the abnormal proliferation and differentiation of cells, thereby promoting malignant transformation. Oncogenic mutations in *CTNNB1*, most frequently missense mutations in exon 3, have been recurrently identified in liver [6], and uterine [7] cancers, and in a small subset of non-small cell lung cancers (NSCLCs) [8–10]. However, the clinical significance of these *CTNNB1* gene mutations in NSCLCs and LUADs is still unclear.

In this present study, we characterize the clinicopathologic characteristics and prognostic implications of oncogenic *CTNNB1* gene mutations in a surgically resected LUAD cohort with next-generation sequencing (NGS) tests. We have further investigated the predictive efficacy of β-catenin immunohistochemistry (IHC) in LUAD in which the *CTNNB1* gene mutation status was compared with the cellular location of β-catenin IHC.

## Materials and methods

### Study population

**Fig 1:**
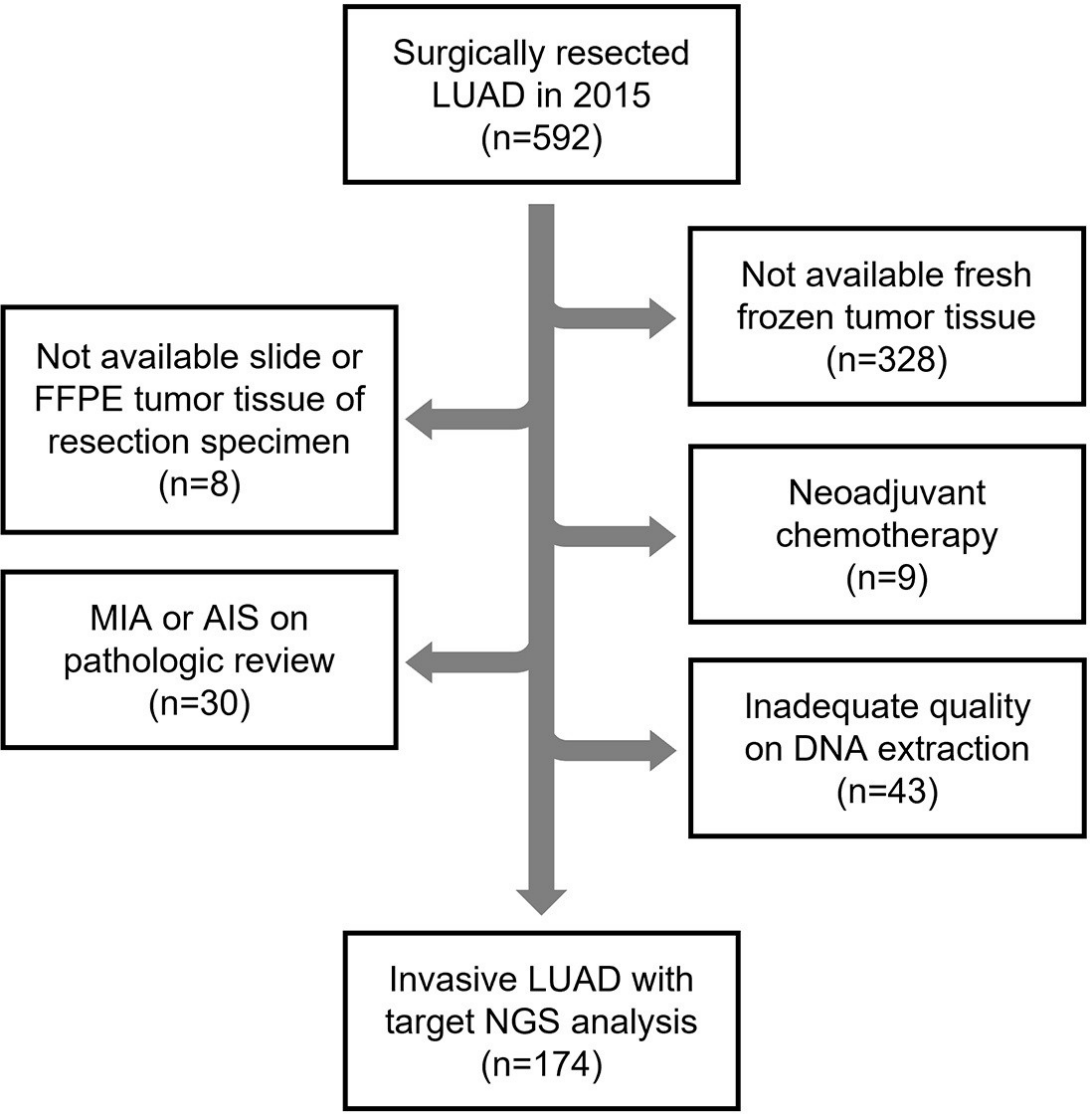
Flow chart depicting the inclusion and exclusion criteria for the present study cohort.

The study was approved by the Institutional Review Board of Asan Medical Center (approval number, 2018–1198). The patient’s informed consent was waived by the Institutional Review Board upon the deidentification process. Initially, we retrieved 592 LUAD patients that had undergone surgical resection from January to December of 2015 at Asan Medical Center. We excluded any patients for whom there were no available fresh-frozen, formalin-fixed paraffin-embedded (FFPE) tumor tissue samples, or histopathologic slides of surgically resected specimens. Patients who had received neoadjuvant therapy or been diagnosed with minimally invasive adenocarcinoma or adenocarcinoma in situ were also excluded. Finally, any cases with nucleic acid samples of inadequate quality for further molecular analysis following DNA extraction were further excluded (Fig 1). A final total of 174 LUAD patients was enrolled in this present study [11]. All available hematoxylin and eosin (H&E)-stained slides derived from the resected tumors were reviewed by three pathologists (Y.K., B.A., and H.S.H.) to re-evaluate the predominant histologic pattern, recently proposed IASLC grade [12], size of the invasive carcinoma component [13], presence of lymphovascular invasion (LVI), and any spread through the alveolar spaces (STAS). We confirmed that the study cohort did not include any cases of low-grade fetal adenocarcinomas, which frequently harbor *CTNNB1* mutations [14]. All of our current study cases were re-staged in accordance with the 8th edition of the AJCC Cancer Staging Manual [15].

### Genomic analysis

Targeted next-generation sequencing was performed for the tumor-only samples of the enrolled cases using AMC OncoPanel version 4.2, as described previously [11]. Briefly, the tumor area of each sample was bluntly dissected from sections of the tumor tissue blocks and the DNA was extracted and assessed for its quality. Subsequently, the samples that were appropriate for further analysis were subjected to sequencing using NextSeq 550Dx (Illumina Inc, San Diego, CA). After these bioinformatics analyses, all identified variants were manually reviewed by an experienced pathologist (H.S.H.) using an IGV genomic viewer system [16] to remove false-positive variants. All output files were integrated and visualized using R package *maftools* version 2.6.05 [17].

### Tissue microarray construction

Tissue microarray (TMA) blocks of the tumor tissues were constructed for evaluation of the immunophenotype. Briefly, two to three cores of each tumor tissue were obtained from the FFPE tissue blocks using 2 mm-diameter trephine apparatus, and representative tumor areas were targeted by reviewing the slides by the pathologists. The cores were then serially embedded into the void recipient paraffin blocks. Finally, 8 recipient blocks embedded with 527 tumor tissue cores were produced.

### β-catenin immunohistochemistry

IHC for β-catenin was carried out on the 4 μm-thick sections of the TMA blocks. Briefly, sections of the paraffin blocks were deparaffinized using EZPrep solution (Ventana Medical Systems, Tucson, AZ), and heat-induced epitope retrieval was done under Cell Conditioning 1 buffer (Ventana). After blocking of the endogenous peroxidase under 3% hydrogen peroxide solution and washout, the sections were incubated with diluted anti-β-catenin primary antibody (titer 1:200; clone 14; Cell marque, Rocklin, CA) for 16 minutes. After the incubation with the secondary antibody, the antigen-antibody reaction signal was visualized under the OptiView DAB IHC Detection kit (Ventana). Finally, the sections were counterstained with hematoxylin. The TMA sections stained with β-catenin IHC were carefully reviewed by two pathologists (Y.K. and H.S.H.). Aberrant β-catenin expression was considered to be present when nuclear staining was apparent at any percentage within the examined tumor cells, regardless of cytoplasmic staining (S1 Fig) [18]. Cytoplasmic staining of β-catenin was difficult to reliably discern from normal membranous staining in some circumstances and we therefore did not interpret cases with only cytoplasmic staining as having aberrant β-catenin expression.

### Statistical analysis

The prognostic impact of each gene mutation was evaluated using the *survGroup* function of R package *maftools* version 2.6.05 [17], with adjustment for multiple tests using a false discovery rate (FDR), calculated using the R function *p*.*adjust* in accordance with the Benjamin-Hochberg method [19]. The differences in the frequencies of each categorical variable according to the patient subgroup were evaluated using chi-square tests. If the expected frequencies were too small, Fisher’s exact tests were carried out as adjuncts. Differences in continuous variables according to the subgroups were investigated using Mann-Whitney U test or Kruskal-Wallis test when there were two or more subgroups, respectively. Univariate and multivariate survival analysis of the categorical variables were performed using Kapan-Meier and Cox proportional hazard regression, respectively. All of these statistical analyses were carried out using R version 4.0.5 (R Foundation of Statistical Computing, Vienna, Austria). A two-sided P-value <0.05 or FDR <0.1 (in the setting of multiple tests) were considered to indicate statistical significance.

## Results

### Identification of single gene alterations and their prognostic implications

**Fig 2:**
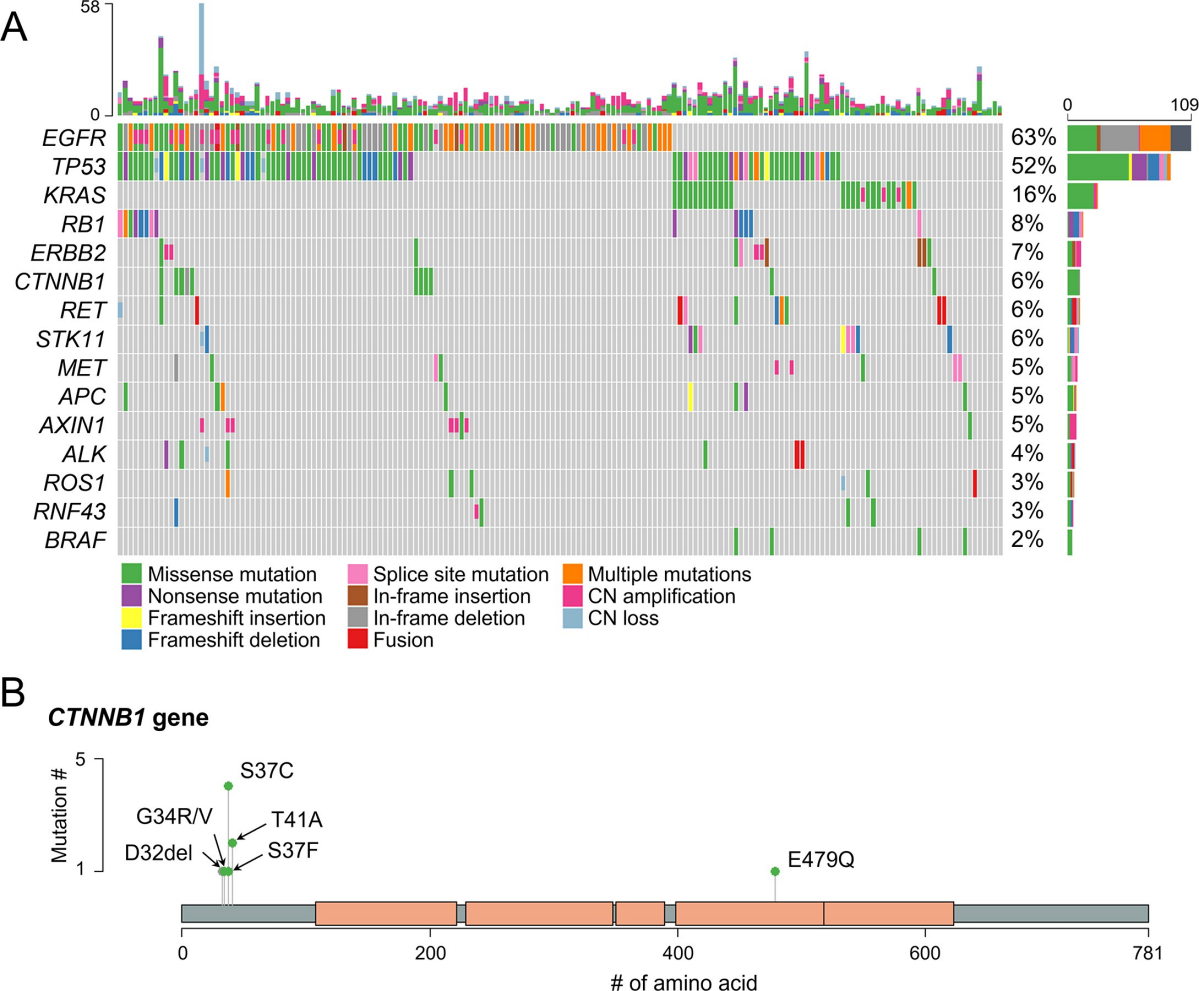
Genomic alterations of Wnt/β-catenin pathway genes identified among the study cohort. (A) Overview of genomic alterations among the whole cohort including common drivers and Wnt/β-catenin pathway genes included in the target next-generation sequencing panel. (B) Lollipop plot of the *CTNNB1* gene showing a predilection for mutations in the exon 3 hotspot region.

The overall genomic alterations identified in the present LUAD study group are presented in Fig 2A. Variations in the *EGFR* gene were the most common, occurring in 63% of the cases, including one case with amplification. *TP53* and *KRAS* mutations were identified in 52% and 16% of the cases, respectively. Oncogenic fusions of the *ALK*, *ROS1*, and *RET* genes were rare in our present cohort, and were present in only 2 (1.1%), 1 (0.6%), and 4 cases (2.3%), respectively. Mutations of Wnt/β-catenin pathway genes were identified in 24 cases (13.8%). Of these, mutations in the *CTNNB1* gene were the most frequently observed and were detected in 11 cases (6.3%). This was followed by mutations in the *APC* (8 cases, truncated mutation in 3 cases), *RNF43* (4 cases, truncated mutation in 1 case), and *AXIN1* (2 cases) genes.

To identify gene mutations that had a significant survival impact among our LUAD patients, the genomic alteration status of all genes showing variations in 5 or more cases was analyzed in accordance with OS and RFS outcomes. During the analysis, we excluded 5 patients who were treated post-operatively with EGFR TKI because of the pleural seeding identified during the operation (n = 169). These results are presented in Table 1. Only *TP53* and *MSH6* gene alterations were significantly associated with poorer OS and RFS outcomes, respectively (FDR<0.1). A *CTNNB1* gene alteration was associated with a poor RFS, but non-significantly (FDR>0.1). Nevertheless, the same analysis within the *EGFR*-mutant subgroup (n = 100) revealed that a *CTNNB1* gene alteration along with an *RB1* mutation was also significantly correlated with a poorer RFS. An *RB1* gene alteration was also significantly associated with poor OS (Table 2). To exclude the possibility that passenger mutations of unknown significance had been included in these assessments, we further characterized the oncogenic potential of the gene alterations. We found that 98% (89/91) of the *TP53* and 93% (13/14) of the *RB1* gene alterations were annotated as oncogenic or likely oncogenic in the OncoKB database [20]. Furthermore, 91% (10/11) of the *CTNNB1* mutations were in the exon 3 region, a known mutation hotspot [21], and were also annotated as oncogenic or likely oncogenic in the OncoKB database (Fig 2B). However, none of the *MSH6* gene alterations were annotated as oncogenic or likely oncogenic. These results suggest that *TP53*, *RB1*, and *CTNNB1* gene alterations have a significant influence on the survival outcomes of LUAD patients.

**Table 1:** Gene alterations found to be associated with overall and recurrence-free survival outcomes in the entire cohort (n = 169).

Overall survival						Recurrence-free survival					
Genes	# of altered cases		HR	*P*	FDR	Genes	# of altered cases		HR	*P*	FDR
	Entire	Oncogenic					Entire	Oncogenic			
***TP53***	**91**	**89**	**3.46**	**0.0001**	**0.012**	***MSH6***	**6**	**0**	**4.21**	**0.0003**	**0.031**
*MSH6*	6	0	4.28	0.003	0.145	*TP53*	91	89	2.10	0.002	0.103
*POLE*	7	0	3.92	0.005	0.190	*KDR*	7	2	4.16	0.003	0.103
*AXL*	9	4	3.08	0.007	0.198	*HGF*	9	0	3.06	0.006	0.133
						*POLE*	7	0	3.34	0.006	0.133
						*CTNNB1*	11	10	2.63	0.008	0.143

Genes altered in at least five cases were included in these analyses. Bolding denotes statistical significance (FDR<0.1)

Abbreviations: FDR, false discovery rate; HR, hazard ratio.

**Table 2:** Gene alterations found to be associated with overall and recurrence-free survival outcomes in the *EGFR*-mutant subgroup (n = 100).

Overall survival						Recurrence-free survival					
Genes	# of altered cases		HR	*P*	FDR	Genes	# of altered cases		HR	*P*	FDR
	Entire	Oncogenic					Entire	Oncogenic			
***RB1***	**7**	**6**	**8.76**	**<0.0001**	**<0.0001**	***RB1***	**7**	**6**	**5.83**	**<0.0001**	**0.0004**
*ATM*	6	0	4.14	0.005	0.105	***CTNNB1***	**8**	**8**	**4.16**	**0.0002**	**0.005**
*MED12*	5	3	4.58	0.007	0.105						
*AXL*	6	1	3.66	0.013	0.133						
*TP53*	53	52	2.73	0.015	0.133						

Genes altered in at least five cases were included in these analyses. Bolding denotes statistical significance (FDR<0.1)

Abbreviations: FDR, false discovery rate; HR, hazard ratio.

### Clinicopathologic features of lung adenocarcinomas cases with an altered Wnt/β-catenin pathway

The clinicopathologic features of the entire cohort (n = 174) are presented in Table 3. Ninety-five (54.6%) of the current study patients were men with a mean age of 63.3 years (range, 32–83 years). About half of the patients (n = 88) were never-smokers, while 48 (27.6%) and 38 (21.8%) patients were previous and current smokers, respectively. Ninety-seven patients (55.7%) had presented with pathologic stage I disease. Notably, 10 patients had initially presented with stage IV disease, 5 of which were treated with EGFR TKI because of pleural seeding identified in the operation field. An acinar-predominant pattern (39.1%) was the most frequent histologic pattern observed. About 25% of the patients received an adjuvant treatment because of a locally advanced disease or due to the presence of LVI, which included platinum doublet chemotherapy (13.2%), chemoradiation therapy (9.8%), and radiation therapy (3.4%). In the comparison of clinicopathologic characteristics, stratified according to the Wnt/β-catenin pathway gene alteration status (oncogenic *CTNNB1* mutation [*CTNNB1*-ONC, n = 10], Wnt/β-catenin gene alterations other than *CTNNB1* [Wnt/β-catenin-OTHER, n = 14], and wild type (WT) for Wnt/β-catenin pathway genes [Wnt/β-catenin-WT, n = 150]), LVI was more frequent in the *CTNNB1*-ONC and Wnt/β-catenin-OTHER groups with statistical significance (*P* = 0.002). The *CTNNB1*-ONC group showed STAS more frequently compared to the Wnt/β-catenin-OTHER and WT groups, with marginal significance (*P* = 0.074). The frequencies of IASLC grade 3 and *EGFR* gene mutations were also found to be increased in the *CTNNB1*-ONC group with marginal significance (*P* = 0.067). None of the *CTNNB1*-ONC LUADs displayed *KRAS* or *RB1* alterations. By histologic examination, oncogenic *CTNNB1*-altered LUADs generally displayed the features of conventional non-mucinous adenocarcinoma with predominant acinar (4/10), papillary (4/10), or solid (2/10) patterns. However, no significant histologic differences were found between *CTNNB1*-ONC, Wnt/β-catenin-OTHER, and Wnt/β-catenin-WT LUADs.

**Table 3:** Clinicopathologic features of the lung adenocarcinoma patients according to the presence of Wnt/β-catenin pathway gene alterations.

		Wnt/β-catenin pathway genes			Total (n = 174)	*P*
		*CTNNB1*-ONC (n = 10)	Wnt/β-catenin-OTHER (n = 14)	Wnt/β-catenin-WT (n = 150)		
Age (mean ± SD)		63.8 ± 10.5	65.4 ± 7.9	63.1 ± 10.8	63.3 ± 10.5	0.835
Gender	Male	4 (40.0)	9 (64.3)	82 (55.5)	95 (54.7)	0.505
	Female	6 (60.0)	5 (35.7)	68 (44.5)	79 (45.3)	
Smoking history	Never	6 (60.0)	5 (35.7)	77 (51.3)	88 (50.6)	0.516
	Previous	1 (10.0)	5 (35.7)	42 (28.0)	48 (27.6)	
	Current	3 (30.0)	4 (28.6)	31 (20.7)	38 (21.8)	
Pathologic stage	IA/B	4 (40.0)	7 (50.0)	84 (56.8)	95 (54.6)	0.353
	IIA/B	5 (50.0)	4 (28.6)	27 (18.2)	36 (20.7)	
	IIIA/B/C	1 (10.0)	2 (14.3)	28 (18.9)	31 (17.8)	
	IVA/B	0	1 (7.1)	9 (6.1)	10 (5.7)	
	Not available	0	0	2 (1.3)	2 (1.1)	
Procedure	Wedge resection	1 (10.0)	2 (14.3)	15 (10.0)	18 (10.3)	0.386
	Segmentectomy	1 (10.0)	1 (7.1)	7 (4.7)	9 (5.2)	
	Lobectomy	8 (80.0)	10 (71.4)	125 (83.3)	143 (82.2)	
	Bilobectomy	0	0	2 (1.3)	2 (1.1)	
	Pneumonectomy	0	1 (7.1)	1 (0.7)	2 (1.1)	
Pleural invasion	Absent	8 (80.0)	9 (64.3)	96 (64.0)	113 (64.9)	0.647
	PL1	0	2 (14.3)	28 (18.7)	30 (17.2)	
	PL2	2 (20.0)	2 (14.3)	15 (10.0)	19 (10.9)	
	PL3	0	1 (7.1)	11 (7.3)	12 (6.9)	
Histologic type	Lepidic	0	0	4 (2.7)	4 (2.3)	0.997
	Acinar	4 (40.0)	6 (42.9)	58 (38.7)	68 (39.1)	
	Papillary	4 (40.0)	4 (28.6)	34 (22.7)	42 (24.1)	
	Cribriform	0	0	5 (3.3)	5 (2.9)	
	Micropapillary	0	0	6 (4.0)	6 (3.4)	
	Solid	2 (20.0)	3 (21.4)	29 (19.3)	34 (19.5)	
	Mucinous	0	1 (7.1)	14 (9.3)	15 (8.6)	
IASLC grade	Grade 1	0	0	6 (4.0)	6 (3.4)	0.753
	Grade 2	2 (20.0)	3 (21.4)	50 (33.3)	55 (31.6)	
	Grade 3	8 (80.0)	11 (78.6)	94 (62.7)	113 (64.9)	
LVI	Absent	2 (20.0)	2 (14.3)	82 (54.7)	86 (49.4)	0.002
	Present	8 (80.0)	12 (85.7)	68 (45.3)	88 (50.6)	
STAS	Absent	0	4 (28.6)	50 (33.3)	54 (31.0)	0.074
	Present	10 (100.0)	10 (71.4)	100 (66.7)	120 (69.0)	
*EGFR* mutation	Absent	2 (20.0)	9 (64.3)	58 (38.7)	69 (39.7)	0.067
	Present	8 (80.0)	5 (35.7)	92 (61.3)	105 (60.3)	
*KRAS* mutation	Absent	10 (100.0)	11 (78.6)	129 (86.0)	150 (86.2)	0.379
	Present	0	3 (21.4)	21 (14.0)	24 (13.8)	
*TP53* alteration	Absent	5 (50.0)	7 (50.0)	71 (47.3)	83 (47.7)	1.000
	Present	5 (50.0)	7 (50.0)	79 (52.6)	91 (52.3)	
*RB1* alteration	Absent	10 (100.0)	11 (78.6)	140 (92.1)	161 (93.3)	0.126
	Present	0	3 (21.4)	10 (7.9)	13 (6.7)	
Post-operative treatment	None	6 (60.0)	10 (71.4)	107 (71.3)	123 (70.7)	0.128
	Chemoradiation	1 (10.0)	1 (7.1)	15 (10.0)	17 (9.8)	
	Chemotherapy	1 (10.0)	3 (21.4)	19 (12.7)	23 (13.2)	
	Radiation	2 (20.0)	0	4 (2.7)	6 (3.4)	
	EGFR TKI	0	0	5 (3.3)	5 (2.9)	

Abbreviations: LVI, lymphovascular invasion; STAS, spread through alveolar space; TKI, tyrosine kinase inhibitor; WT, wild type.

### β-catenin immunohistochemistry staining patterns and their association with the Wnt/β-catenin pathway mutation status

**Fig 3:**
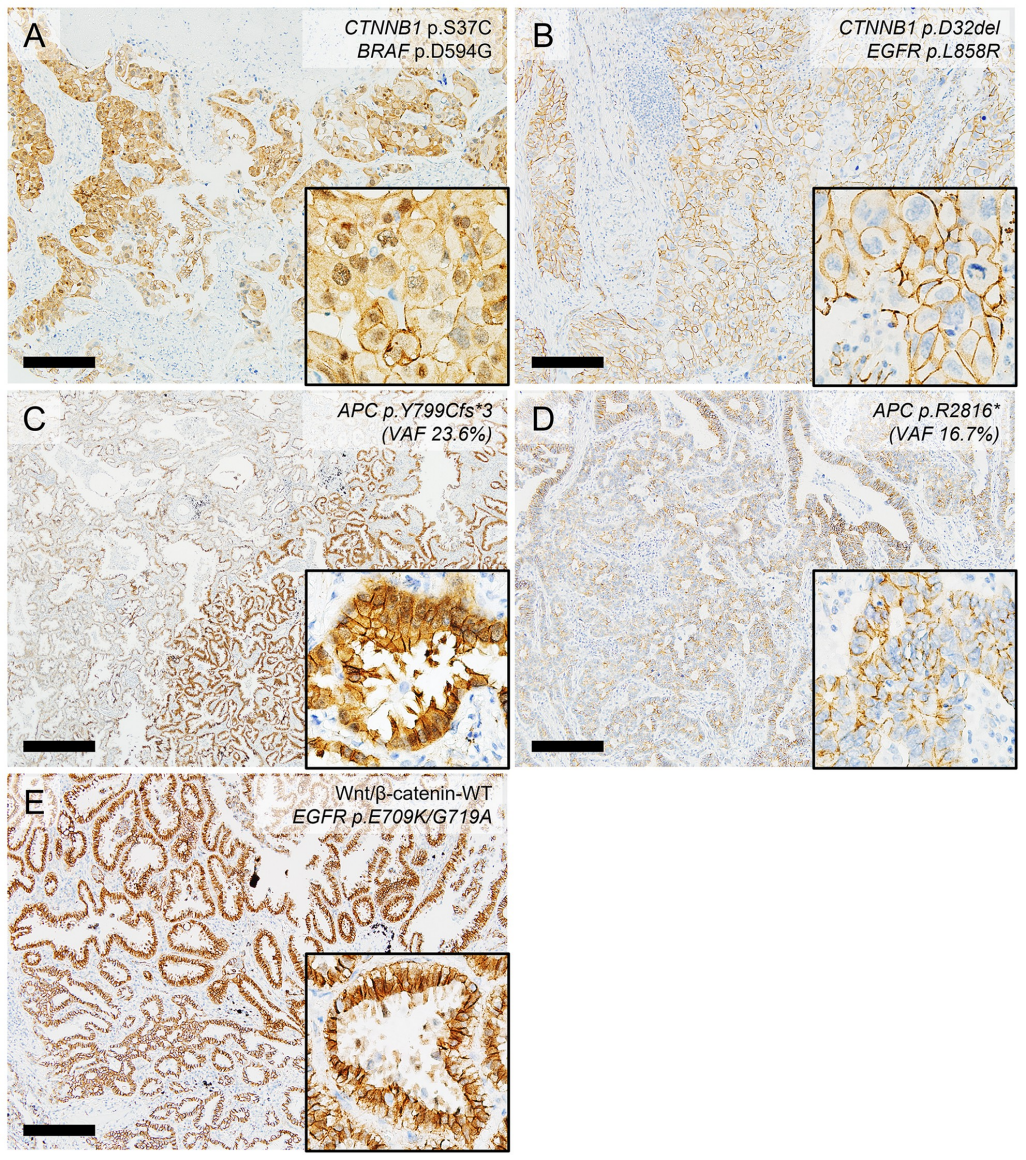
Correlation between β-catenin immunostaining patterns and Wnt/β-catenin pathway alterations. (A) Aberrant nuclear β-catenin staining in a case harboring an oncogenic *CTNNB1* mutation (p.S37C). (B) Another patient showing an oncogenic *CTNNB1* mutation (p.D32del) with absent nuclear staining. (C,D) Two cases had truncated *APC* mutations (C, p.Y799Cfs*3; D, p.R2816*), however aberrant nuclear β-catenin staining was only evident in case (C). (E) The A sample with no genomic alterations in the Wnt/β-catenin pathway displayed aberrant nuclear β-catenin staining, and these discrepancies were noted in 6.0% of the current study cases. All scale bars, 200 μm.

IHC for β-catenin on the LUAD TMA sections revealed aberrant nuclear staining in 17 of 174 cases (9.8%). Aberrant β-catenin expression was found to be significantly correlated with the Wnt/β-catenin pathway alteration status, which was most frequent in the *CTNNB1*-ONC group (*P* < 0.0001; Fig 3A). Nevertheless, only 4 out of 10 *CTNNB1*-ONC cases (40%) showed aberrant β-catenin expression (Table 4 and Fig 3B). Additionally, only 2 out of 14 cases (14.3%) in the Wnt/β-catenin-OTHER group showed aberrant β-catenin expression. Surprisingly, aberrant β-catenin expression was identified in only in 1 of the 3 cases identified with a truncated *APC* gene mutation (Fig 3C and 3D). In addition, aberrant β-catenin expression was identified in 9 out of 150 (6.0%) cases in the Wnt/β-catenin-WT group (Table 4 and Fig 3E). These results suggest that the β-catenin IHC status can show discrepancies with the actual mutation status of Wnt/β-catenin pathway genes in LUAD tissues.

**Table 4:** Comparisons of the β-catenin immunohistochemistry status with Wnt/β-catenin pathway gene alterations.

		Wnt/β-catenin pathway status			Total (n = 174)
		*CTNNB1*-ONC (n = 10)	Wnt/β-catenin-OTHER (n = 14)	Wnt/β-catenin-WT (n = 150)	
β-catenin IHC status	Aberrant	6 (60.0)	2 (14.3)	9 (6.0)	17 (9.8)
	Normal	4 (40.0)	12 (85.7)	141 (94.0)	157 (90.2)

*Fisher’s exact test *P* < 0.0001

### Survival analysis in accordance with the Wnt/β-catenin pathway mutation status and β-catenin immunophenotype of the lung adenocarcinoma

**Fig 4:**
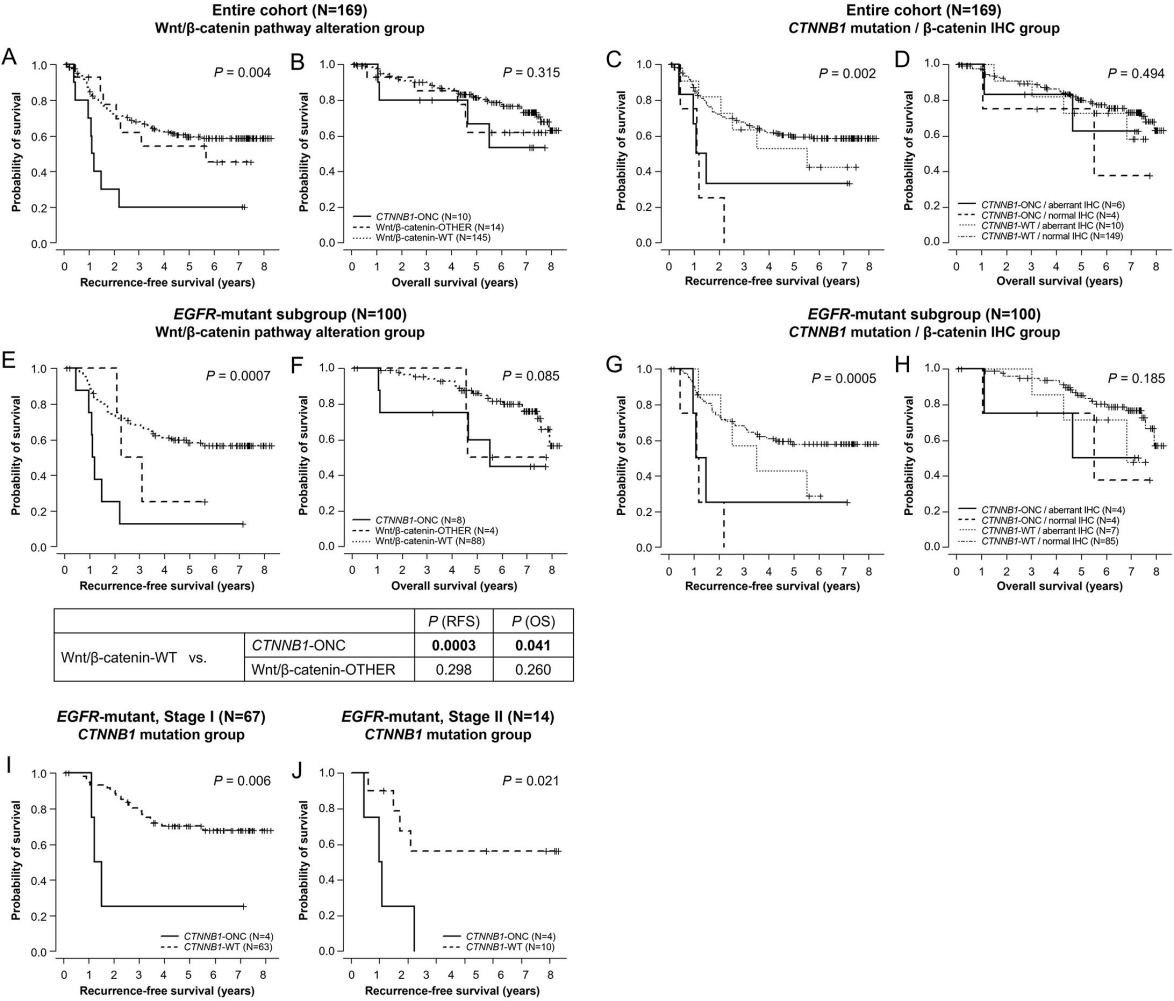
Kaplan-Meier survival curves in accordance with Wnt/β-catenin pathway alterations and the β-catenin IHC status. (A) Recurrence-free survival (RFS) in the *CTNNB1*-ONC group was significantly poorer than that in the Wnt/β-catenin-OTHER and -WT groups. No significant difference in the RFS rate between the Wnt/β-catenin-OTHER and -WT cases was observed. (B) Overall survival (OS) was not significantly different between the Wnt/β-catenin pathway groups. (C) RFS outcomes of the *CTNNB1*-ONC and *CTNNB1*-WT groups (combination of Wnt/β-catenin-OTHER and -WT groups) stratified by the β-catenin IHC status. The *CTNNB1*-ONC group was significantly associated with a poor RFS regardless of the β-catenin IHC status. It was noteworthy that there was no significant difference in the RFS in accordance with the β-catenin IHC status in the *CTNNB1*-WT group. (D) As shown in (B), no significant difference in OS outcome was found between patients with a different *CTNNB1* or β-catenin IHC status. (E, F) RFS (E) and OS (F) outcomes in the *CTNNB1*-ONC group were significantly poorer than those in the Wnt/β-catenin-WT group. The Wnt/β-catenin-OTHER group also displayed a poorer RFS and OS than the Wnt/β-catenin-WT group, but without statistical significance. (G) Similar to the findings for the entire cohort, the β-catenin IHC status did not show any significant association with the RFS outcomes of the *CTNNB1*-ONC and *CTNNB1*-WT groups within the *EGFR*-mutant subgroup. (H) No significant differences in OS outcomes were evident in accordance with the *CTNNB1* or β-catenin IHC status in the *EGFR*-mutant subgroup. (I-J) The *CTNNB1*-ONC group was significantly correlated with a poorer RFS both in the stage I (I) and stage II (J) subgroups of *EGFR*-mutant LUAD cases.

Univariate survival analysis results for the entire LUAD patients in accordance with the Wnt/β-catenin pathway alteration and β-catenin IHC findings are presented in Fig 4A–4D. Survival analysis was conducted after excluding 5 patients treated with EGFR TKI, in order to exclude any prognostic effect of this treatment. The *CTNNB1*-ONC group was significantly associated with a poorer RFS compared to the other groups (Fig 4A). Notably however, the OS outcomes among the *CTNNB1*-ONC group patients did not significantly differ from the Wnt/β-catenin-WT or Wnt/β-catenin-OTHER groups (Fig 4B). Similar to the Wnt/β-catenin gene status, neither the RFS nor OS outcomes were significantly different according to the β-catenin immunophenotype within *CTNNB1*-ONC and *CTNNB1*-WT (combination of Wnt/β-catenin-WT and OTHER groups) groups (Fig 4C and 4D). RFS analysis in accordance with the stratified *CTNNB1*-ONC and *CTNNB1*-WT subgroups by *TP53* alteration revealed that a *TP53* alteration was significantly associated with a poor RFS in the *CTNNB1*-WT group, but that no significant RFS difference was evident with this variation within the *CTNNB1*-ONC group (S2 Fig).

We conducted univariate survival analysis using a similar scheme for both the *EGFR*-mutant (n = 100) (Fig 4E and 4H) and *EGFR*-WT (n = 69) (S2 Fig) subgroups. As stated previously, 5 *EGFR*-mutant cases treated with EGFR TKI were excluded. In the analysis of the *EGFR*-mutant subgroup, the *CTNNB*-ONC group displayed both a poorer RFS and OS compared to the Wnt/β-catenin-WT group (RFS, *P* = 0.0003; for OS, *P* = 0.041). By contrast, the Wnt/β-catenin-WT-OTHER group showed no significant survival difference (*P* > 0.05) (Fig 4E and 4F). Similarly to the findings for the whole cohort, the β-catenin IHC results were not significantly associated with the OS or RFS outcomes within the *CTNNB1*-ONC and *CTNNB1*-WT groups of *EGFR*-mutant cases (Fig 4G and 4H). The *CTNNB1*-ONC group also exhibited a significantly poorer RFS in both stage I (*P* = 0.006) (Fig 4I) and stage II (*P* = 0.021) (Fig 4J) patients in further subgroup analysis of *EGFR*-mutant cases. Furthermore, multivariate survival analysis revealed that *CTNNB1*-ONC was significantly associated with a poor RFS in the *EGFR*-mutant subgroup, even after adjusting for the AJCC pathologic stage, IASLC grade, presence of LVI, STAS, and *RB1* mutation (hazard ratio 5.98, 95% confidence interval 2.03–17.61, *P* = 0.001; Table 5). In contrast to the univariate analysis results, the Wnt/β-catenin gene alteration status did not show a significant association with the OS rates by multivariate analysis. In addition, none of the Wnt/β-catenin mutation group or β-catenin IHC findings had a significant correlation with the survival outcomes in the *EGFR*-wild type subgroup (S2 Fig).

**Table 5:** Results of Cox proportional hazard analysis in the *EGFR*-mutant subgroup (n = 98).

Parameters		Overall survival			Recurrence-free survival		
		HR	95% CI	*P*	HR	95% CI	*P*
Wnt/β-catenin alteration status	Wnt/β-catenin-WT	1	-	-	1	-	-
	*CTNNB1*-ONC	2.82	0.66–12.14	0.154	5.98	2.03–17.61	0.001
	Wnt/β-catenin-OTHER	1.06	0.14–7.88	0.204	1.19	0.30–4.63	0.806
Pathologic Stage	Stage I	1	-	-	1	-	-
	Stage II	5.59	1.60–19.50	0.007	1.35	0.53–3.43	0.529
	Stage III-IV	7.37	2.21–24.57	0.001	4.51	2.09–9.72	0.0001
IASLC grade	Grade 1–2	1	-	-	1	-	-
	Grade 3	4.02	0.84–19.24	0.082	2.72	1.13–6.59	0.026
Presence of LVI		0.87	0.27–2.85	0.847	2.20	0.98–4.98	0.057
Presence of STAS		2.01	0.53–7.61	0.303	0.69	0.30–1.62	0.395
Presence of *RB1* alteration		5.41	1.45–20.15	0.012	3.34	1.23–9.08	0.018

Abbreviations: CI, confidence interval; HR, hazard ratio; LVI, lymphovascular invasion; STAS, spread through alveolar space; WT, wild type.

## Discussion

Our present results demonstrated that oncogenic *CTNNB1* mutations were present in 5.7% of the surgically resected LUAD cases included in our current study cohort. These cases harboring an oncogenic *CTNNB1* mutation were only associated with an increased frequency of LVI and showed no significant correlation with any other clinicopathologic characteristics. Cases with oncogenic *CTNNB1* mutations also showed a significant association with a poorer RFS outcome, which was pronounced in the *EGFR*-mutant but not in the *EGFR*-wild type subgroup. These findings were further confirmed by multivariate survival analysis. Hence, a *CTNNB1* gene mutation could be a useful indicator of a post-operative recurrence of *EGFR*-mutant LUAD and may require routine evaluation.

*CTNNB1* gene mutations are known to be found in 2–3% of NSCLCs [22], including TCGA-LUAD cases (2.6%, 13/507) [23]. This is a lower incidence than that observed in our present study cohort and this discrepancy could be attributable to a disparate frequency of *EGFR* gene mutations between populations. de Montpréville and colleagues have reported previously that *CTNNB1* gene mutations are more prevalent in cases with *EGFR* gene mutations also [24], which is in line with our current observations. Because our present cohort harbored more *EGFR*-mutant cases than previously reported Western populations, it is not surprising that we detected a higher frequency of *CTNNB1* mutations. Notably, another previous study involving a Chinese LUAD cohort also found a similar *CTNNB1* mutation frequency (5.3%) to that in our current series [25].

The prognostic implications of *CTNNB1* mutations in LUAD have not been widely investigated to date. Several studies of *EGFR*-mutant LUADs treated with tyrosine kinase inhibitors suggested a negative prognostic impact of *CTNNB1* mutations [26–28], but this possibility has remained under debate [24]. A further report evaluating the prognostic impact of *CTNNB1* mutations in a large cohort of surgically resected LUADs showed a trend only toward a poorer OS without statistical significance [25]. We find from our current analyses however that oncogenic *CTNNB1* mutations are significantly associated with a poor RFS outcome in *EGFR*-mutant LUAD, which implicates these variations as an indicator of higher post-operative recurrence among surgically resected *EGFR*-mutant LUAD cases. However, we could not fully confirm a significant relationship between the presence of an oncogenic *CTNNB1* mutation and OS outcomes in neither *EGFR*-mutant nor *EGFR*-wild type LUAD. Further well-controlled studies will be essential to validate the prognostic significance of *CTNNB1* mutations following a surgical resection in LUAD patients.

Several studies have now indicated that the abnormal cellular localization of β-catenin, a factor thought to indicate Wnt/β-catenin signaling activation [29], is associated with a poorer prognosis in NSCLC [30–37]. These prior reports also considered the detection of cytoplasmic staining in β-catenin IHC experiments as indicative of aberrant expression. However, the nuclear translocalization of β-catenin is known to be essential for the activation of Wnt/β-catenin signaling [38–40], suggesting that a higher level of cytoplasmic β-catenin would have insufficient effects on this activation. In support of this, a prior study has correlated *CTNNB1* mutation and β-catenin IHC patterns in endometrial cancer and found that these mutations were present in only 15% of the cases with cytoplasmic β-catenin staining [18]. Further studies that conduct correlations between β-catenin staining patterns and the Wnt/β-catenin pathway activation status are required to characterize the biologic significance of higher cytoplasmic β-catenin expression in NSCLCs.

We here observed a significant level of discordance between nuclear β-catenin staining and the presence of an oncogenic *CTNNB1* mutation in LUAD. Previous reports that have evaluated the diagnostic accuracy of β-catenin IHC findings in predicting a *CTNNB1* mutation in endometrial cancer showed a high sensitivity and specificity [18, 41]. Nevertheless, a further study of *CTNNB1*-mutated NSCLCs reported a heterogeneity of aberrant β-catenin staining within the same tumor, which casts doubt on the efficacy of β-catenin IHC as a predictor of a *CTNNB1* mutation in NSCLC [24]. Furthermore, our current findings suggest that aberrant β-catenin staining can be observed in LUADs with no alterations in *CTNNB1* or other Wnt/β-catenin pathway genes. We speculate therefore that β-catenin IHC pattern is not a useful predictor of Wnt/β-catenin pathway gene mutations. Also, our present data suggest the possibility that the biologic mechanisms which can activate Wnt/β-catenin signaling other than a *CTNNB1* mutation [42] may not have a prognostic role in *EGFR*-mutant LUADs.

The present study had several limitations of note. First, it was based on a retrospective case-controlled cohort which has an inherent risk of a selection bias. Even though the contributing pathologists diligently selected the most representative tumor areas for next-generation sequencing analysis, the possibility exists that these tests could not identify all subclonal mutations outside the submitted tumor area. Finally, the β-catenin immunophenotypes in our present series were investigated using TMA blocks, which may not fully encompass intratumoral heterogeneity [43].

In conclusion, oncogenic *CTNNB1* mutations can arise in up to 5% of Asian LUAD cases and are associated with a poorer RFS in *EGFR*-mutant LUAD. This suggests that the *CTNNB1* mutation status could serve as a prognostic marker for a higher post-operative recurrence in these cancers. Moreover, β-catenin IHC testing of tumor sections may be inadequate as a surrogate marker of *CTNNB1* gene mutations in LUAD.

## Supporting information

S1 Fig Representative examples β-catenin immunohistochemical staining patterns. (A) Aberrantly diffuse and strong nuclear and cytoplasmic staining and (B) occasional weak nuclear and cytoplasmic staining (white arrows) for β-catenin was observed in the tumor cells. (C) Normal membranous staining of the tumor cells.
(TIF)

S2 Fig (A) Kaplan Meier curve of recurrence-free survival (RFS) in accordance with the CTNNB1 mutation and TP53 alteration statuses within the entire cohort (n = 169). This analysis indicated an invariably poor RFS within the CTNNB1-ONC group regardless of the TP53 alteration status. (B-E) Kaplan-Meier curves of (B and D) RFS and (C and E) overall survival (OS) outcomes according to the (B, C) Wnt/β-catenin pathway alteration status and (D, E) CTNNB1 mutation status, stratified by the β-catenin IHC pattern analyzed within the EGFR-wild type subgroup (n = 69). None of these comparisons were statistically significant.
(TIF)
