# Supplementary figures and images for: An oncogenic CTNNB1 mutation is predictive of post-operative recurrence-free survival in an EGFR-mutant lung adenocarcinoma
Source: PLoS One. 2023 Jun 22;18(6):e0287256. doi: 10.1371/journal.pone.0287256 (PMC10286999; doi:10.1371/journal.pone.0287256)

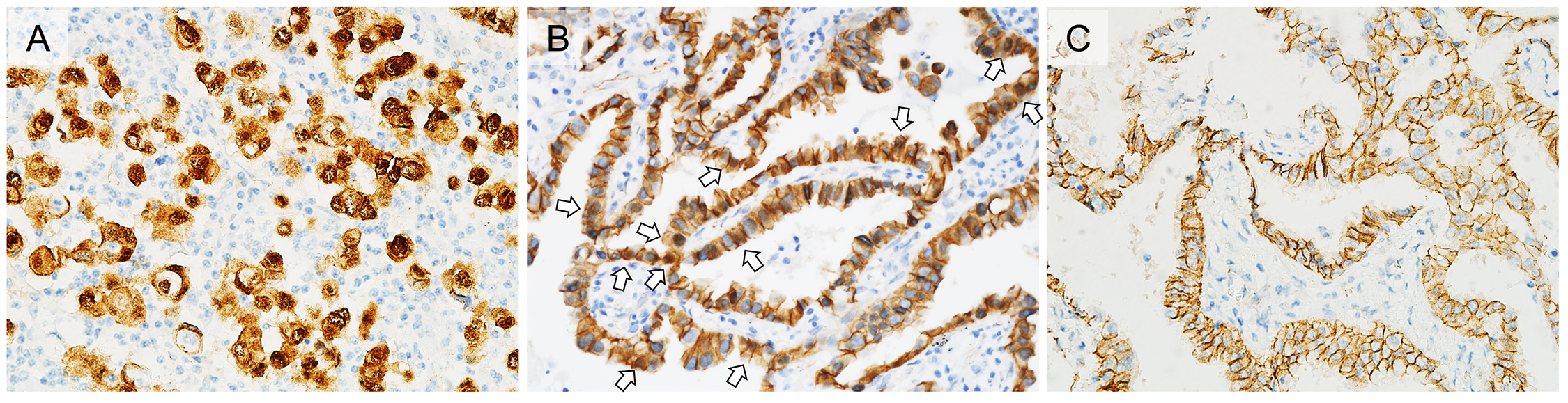

Supplement: S1 Fig — (A) Aberrantly diffuse and strong nuclear and cytoplasmic staining and (B) occasional weak nuclear and cytoplasmic staining (white arrows) for β-catenin was observed in the tumor cells. (C) Normal membranous staining of the tumor cells. (TIF) [file pone.0287256.s001.tif]

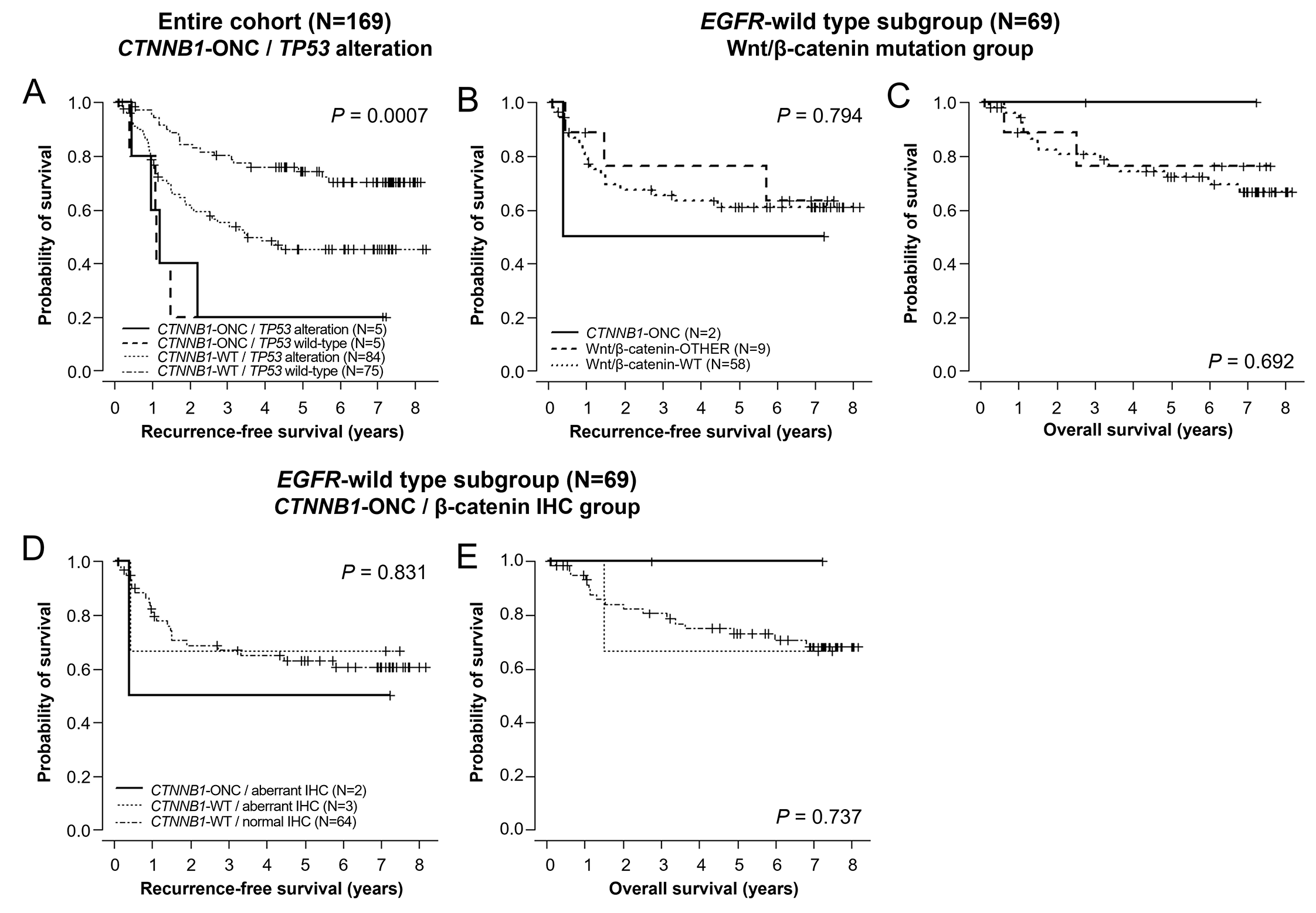

Supplement: S2 Fig — (A) Kaplan Meier curve of recurrence-free survival (RFS) in accordance with the CTNNB1 mutation and TP53 alteration statuses within the entire cohort (n = 169). This analysis indicated an invariably poor RFS within the CTNNB1-ONC group regardless of the TP53 alteration status. (B-E) Kaplan-Meier curves of (B and D) RFS and (C and E) overall survival (OS) outcomes according to the (B, C) Wnt/β-catenin pathway alteration status and (D, E) CTNNB1 mutation status, stratified by the β-catenin IHC pattern analyzed within the EGFR-wild type subgroup (n = 69). None of these comparisons were statistically significant. (TIF) [file pone.0287256.s002.tif]
